# Therapeutic Doses of Nonsteroidal Anti-Inflammatory Drugs Inhibit Osteosarcoma MG-63 Osteoblast-Like Cells Maturation, Viability, and Biomineralization Potential

**DOI:** 10.1155/2013/809891

**Published:** 2013-09-19

**Authors:** E. De Luna-Bertos, J. Ramos-Torrecillas, O. García-Martínez, A. Guildford, M. Santin, C. Ruiz

**Affiliations:** ^1^Department of Nursing, Faculty of Health Sciences, University of Granada, Avenida Madrid s/n, 18071 Granada, Spain; ^2^School of Pharmacy & Biomolecular Science, University of Brighton, Huxlye Building, Moulsecoomb, Brighton BN2 4G1, UK; ^3^Institute of Neurosciences, Faculty of Medicine, University of Granada, Granada Health-Science Technology Park, 18100 Armilla, Granada, Spain

## Abstract

Nonsteroidal anti-inflammatory drugs (NSAIDs) are frequently used to reduce pain and inflammation. However, their effect on bone metabolisms is not well known, and results in the literature are contradictory. The present study focusses on the effect of dexketoprofen, ketorolac, metamizole, and acetylsalicylic acid, at therapeutic doses, on different biochemical and phenotypic pathways in human osteoblast-like cells. Osteoblasts (MG-63 cell line) were incubated in culture medium with 1–10 *μ*M of dexketoprofen, ketorolac, metamizole, and acetylsalicylic acid. Flow cytometry was used to study antigenic profile and phagocytic activity. The osteoblastic differentiation was evaluated by mineralization and synthesis of collagen fibers by microscopy and alkaline phosphatase activity (ALP) by spectrophotometric assay. Short-term treatment with therapeutic doses of NSAIDs modulated differentiation, antigenic profile, and phagocyte activity of osteoblast-like cells. The treatment reduced ALP synthesis and matrix mineralization. However, nonsignificant differences were observed on collagen syntheses after treatments. The percentage of CD54 expression was increased with all treatments. CD80, CD86, and HLA-DR showed a decreased expression, which depended on NSAID and the dose applied. The treatments also decreased phagocyte activity in this cellular population. The results of this paper provide evidences that NSAIDs inhibit the osteoblast differentiation process thus reducing their ability to produce new bone mineralized extracellular matrix.

## 1. Introduction

Nonsteroidal anti-inflammatory drugs (NSAIDs) are commonly prescribed by their therapeutic indications (anti-inflammatory, analgesic, and antipyretic) [1, 2]. The clinical effectiveness of NSAIDs is based on the inhibition of cyclooxygenase (COX) activity, leading to diminished prostaglandin production [1, 3, 4]. They are administered postoperatively and posttraumatically to achieve analgesia and to reduce inflammation especially in the bony tissue.

However, over the last two decades, several studies have suggested that NSAIDs interfere with bone healing by suppressing its growth and remodeling. The biochemical and cellular pathways affected by this inhibitory action are not fully understood, and many authors have attributed this effect to an impaired osteoblasts proliferative capacity [5–9]. However, other authors have ascertained that NSAIDs, like aspirin or ibuprofen, did not affect bone cells proliferation capacity at therapeutic doses [10, 11].

Studies have also been focusing on the effect of NSAIDs on osteoblast differentiation. Arpornmaeklong et al. (2008) [12] found that indomethacin and celecoxib inhibit cell growth but have a less clear effect on cell differentiation as determined by alkaline phosphatase and osteocalcin synthesis. The inhibitory role of NSAID on osteoblast proliferation and the increase of cell apoptosis through the cyclooxygenase 2 (COX-2) activity has also suggested a potential therapeutic role of these drugs in cancer treatment [13]. 

However, the process of bone formation and bone remodeling goes beyond osteoblast proliferation and differentiation and these processes are finely regulated by both endogenous and exogenous stimuli. Indeed, osteoblasts differentiation of which is regulated by a large number of paracrine, autocrine, and endocrine factors such us growth factors, hormones and cytokines [14, 15] and it is reported that the activity of these cells is regulated by mechanical stimuli. In turn, osteoblasts participate in bone metabolism also regulating osteoclast activity and recent investigations have highlighted their role in the immune response. This immunological function has been shown to include phagocytic activity, T lymphocyte stimulation, and cytokine synthesis [16–20]. Histological studies have highlighted the ability of osteoblast to present antigens [21], while primary cultures and human osteosarcoma cell line MG-63 studies have clearly indicated the phagocytic capacity of osteoblasts against foreign bodies of different size and chemical source [19, 22–24].

The present study analyses the short-term effect of clinical doses of various types of NSAIDs, dexketoprofen, ketorolac, metamizole and acetylsalicylic acid on cell line osteoblasts, MG-63. Short-term culture conditions were chosen to pinpoint the early biochemical pathways triggered by the drugs that lead to impaired bone metabolism and to determine which phenotype was mainly affected by their presence.

## 2. Material and Methods

### 2.1. Cell Culture

The human MG-63 osteosarcoma cell line was purchased from American Type Cultures Collection (ATCC, Manassas, VA) and maintained as described by De Luna-Bertos et al. (2012) [11] in Dulbecco's Modified Eagle Medium (DMEM; Invitrogen Gibco Cell Culture Products, Carlsbad, CA) with 100 IU/mL penicillin (Lab Roger SA, Barcelona, Spain), 50 *μ*g/mL gentamicin (Braum Medical SA, Jaén, Spain), 2.5 *μ*g/mL amphotericin B (Sigma, St Louis, MO, USA), 1% L-glutamine (u/v) (Sigma, St Louis, MO, USA), 2% HEPES (Sigma, St Louis, MO, USA), and supplemented with 10% (v/v) Fetal Bovine Serum (FBS) (Gibco, Paisley, UK). Cultures were kept at 37°C in a humidified atmosphere of 95% air and 5% CO_2_. Cells were detached from culture flask with a solution of 0.05% trypsin (Sigma, St Louis, MO, USA), and 0.02% ethylenediaminetetraacetic acid (w/v) (EDTA) (Sigma, St Louis, MO, USA) and then washed and suspended in complete culture medium with 10% FBS.

 The cells were treated with different drugs, metamizole, dexketoprofen, ketorolac, or acetylsalicylic acid, purchased from Sigma (Sigma, St Louis, MO, USA), at a dose of 1 or 10 *μ*M or without NSAID as control, during different period of time depending on the assay.

### 2.2. Antigenic Phenotype by Flow Cytometry

Osteoblasts (MG-63 cell line) were previously treated with 1 and 10 *μ*M of dexketoprofen, ketorolac, metamizole, and acetylsalicylic acid for 24 h at 37°C. Cells were then detached from the cultured flask by treatment with 0.4% (w/v) EDTA solution, washed, and suspended in Phosphate-Buffered Saline (PBS) at 2 × 10^4^ cells/mL. Cells were labeled by direct staining with the monoclonal antibodies (MAbs) listed in Table 1. Aliquots of 100 *μ*L of cell suspension were incubated with 10 *μ*L of the appropriate MAb for 30 min at 4°C in the dark. Cells were washed, suspended in 1 mL of PBS, and immediately analyzed in a flow cytometer with diodo laser (FASC Canton II, SE Becton Dickinson, Palo Alto, California, USA) at a wavelength of 488 nm to determine the percentage of fluorescent cells. Untreated cells were used as control group. The percentage of antibody-positive cells was calculated from counts of 2000–3000 cells. At least three experiments were run for each antigen in all cultures.

### 2.3. Phagocytic Activity of MG-63 Lines

Phagocytic activity was studied by flow cytometry. Cultured human MG-63 cells were treated with 1 and 10 *μ*M of dexketoprofen, ketorolac, metamizole, and acetylsalicylic acid. Untreated cells were used as control group. Cells were detached from the culture flask by treatment with a solution of 0.05% (w/v) trypsin and 0.02% (w/v) EDTA, washed, and then suspended in complete culture medium with 10% (v/v) FBS at 2 × 10^4^ cells/mL. Cells were labeled by direct staining with labeled latex beads. One hundred microliters of cell suspension were incubated with 200 *μ*L carboxylated FICT-labeled latex beads of 2 *μ*m of diameter (Sigma Adrich, St Louis, USA) for 30 min at 37°C in darkness. Cells were washed, suspended in 1 mL of PBS, and immediately analyzed in a flow cytometer (FASC Canton II, SE Becton Dickinson, Palo Alto, California, USA). Results were expressed as percentage of cells positive for phagocytosis and mean channel fluorescence, which correlates with the number of phagocytosed particles.

### 2.4. Cell Differentiation Assay

The effect on osteoblast-like cells differentiation was assessed by the evaluation of collagen, ALP levels, and the number of calcium deposits (matrix mineralization) produced by MG-63 cells cultured in nonosteogenic and osteogenic mediums and treated with the different NSAIDs. Cells were seeded in an appropriate cells number in a 6-well plate or 24-well plates, depending on assay (Falcon, Becton Dickinson Labware) and cultured in both mediums at 37°C in a humidified atmosphere of 95% air and 5% CO_2_.

Osteogenic medium which consists of complete medium was supplemented with 5 mM *β*-glycerophosphate and 0.05 mM ascorbic acid. 

#### 2.4.1. Collagen Synthesis

Cells at 1 × 10^4^ cells/mL per well into 24-well plates in nonosteogenic and osteogenic mediums were cultured at different concentrations of NSAIDs. After 24 h of incubation, cells were fixed with 3.7% (w/v) formalin, and later 500 *μ*L of picrosirius red stain (Sirius red F3BA) were added. After incubation for 1 hour at room temperature, wells were washed with distiller water three times. Finally, the samples were analyzed under light microscope (Inverted NIKON Elipse TE2000U).

Also the cellular morphology was studied by scanning electron microscopy (SEM), with the aim of analyzing the fibrin of collagen, after seven days of incubation at different concentration of NSAIDs in both mediums (osteogenic and nonosteogenic). After removing the supernatant, the samples were washed gently with sterile PBS and were fixed with 3.7% formalin for 30 min. After that, dehydrated the cells through a series of graded alcohols and were allowed the samples to air dry in a sterile flow hood. After 24 h, cells were coated with palladium, and finally the samples were analyzed by SEM.

#### 2.4.2. Alkaline Phosphatase Activity

ALP activity was quantified using a colorimetric assay (Diagnostic kit 104-LL, Sigma, St. Louis, MO, USA) to determine early osteoblastic differentiation. The assay measures the conversion of the colourless substrate *p*-nitrophenylphosphate by the enzyme ALP to the yellow product *p*-nitrophenol, with the rate of colour change corresponding to the amount of enzyme present in solution. Standards of *p*-nitrophenol (0–250 *μ*M) were prepared from dilutions of a 1000 *μ*M stock solution and assayed in parallel. The ALP assay was performed as described by Sandrini et al. (2005) [25]. The cells in osteogenic and nonosteogenic mediums with NSAIDs or without drugs (control group) were seeded at 1 × 10^4^ cells/mL per well into 24-well plates and cultured for seven days under standard conditions. Then, cells were lysed with 0.1% (v/v) Triton X-100, at 37°C. The samples were centrifuged at 1500 rpm, and the supernatants were stored at −70°C until used. ALP activity was determined with 50 *μ*L of *p*-nitrophenylphosphate (Sigma, St. Louis, MO, USA) as substrate and 50 *μ*L of cells lysate solution and was incubated at 37°C for 45 min in darkness. The enzymatic reaction was stopped by adding 50 *μ*L of 0.1 M NaOH, and finally, the absorbance was measured at 405 nm with a spectrophotometer (Biotek ELx800). The total protein content was also estimated by a protein assay kit from Bio-Rad Laboratories (Nazareth-Eke, Belgium), based on the Bradford's method. All samples were run in triplicate, and specific ALP activity was expressed in U/mg cellular protein.

#### 2.4.3. Matrix Mineralization

The presence of calcium deposits into the cellular matrix was analysed by Alizarin Red S. MG-63 cells were seeded (5 × 10^4^ cells/mL/well) in a 6-well plate and cultured in osteogenic medium with different concentrations of NSAIDs assayed at 37°C in a humidified atmosphere of 95% air and 5% CO_2_. The medium was replaced after 4 days and then every 3 days. We examined the matrix mineralization of each cell line after 7, 15, and 22 days of culture. Wells were washed with 150 mM sodium chloride, fixed in cold 70% ethanol for 5 min, and rinsed three times with distilled water. Wells were then incubated for 10 min with 1 mL of 2% alizarin red S solution buffered at pH 4 with sodium hydroxide, then rinsed five times with distilled water, and finally washed with PBS to reduce nonspecific staining. Calcium deposits present in the extracellular collagen matrix were coloured red, revealing the mineralization nodules, which were counted under light microscopy. Images of stained cultures were captured with a digital camera. Results are expressed qualitatively based on the number of red nodules observed per well, considering (−) = no nodules, (+) = 5–20 nodules, and (++) => 20 nodules per well.

### 2.5. Cell Viability

Cell viability was assessed by counting cells under fluorescent microscopy. Cells were cultured and treated with the different drugs for 24 h. Hoechst/Propidium Iodide (HPI) staining solution was prepared, mixing 20 L of Hoechst 33342 solution (10 *μ*g/mL^−1^) with 20 *μ*L propidium iodide solution (10 *μ*g/mL^−1^) in 460 *μ*L of culture media. The media contained in the wells was removed, and samples were washed three times with 1 mL of sterile PBS by gently sucking/pipetting action. Clean samples were then stained with 10 *μ*L of HPI staining. The number of alive and apoptotic cells were scored by fluorescence microscopy at 40x magnification; six different fields per sample were counted. The data were expressed as mean value.

### 2.6. Statistical Analysis

SPSS version 17.0 (SPSS, Chicago, IL) was used for the data analysis. Mean values (±standard deviation) were calculated for each variable. Analysis of variance (ANOVA) was performed to examine the effects on ALP synthesis considering treatment (dexketoprofen, ketorolac, metamizole, or acetylsalicylic acid) and concentration (1 and 10 *μ*M) compared with control group. Antigenic profile and phagocytic activity were compared using the Student's *t*-test. *P* < 0.05 was considered statistically significant in all tests. At least three experiments were performed in all assays. Data were expressed as mean ± standard deviation (SD).

## 3. Results and Discussion

### 3.1. Effect of NSAID on Antigenic Phenotype

**Figure 1:**
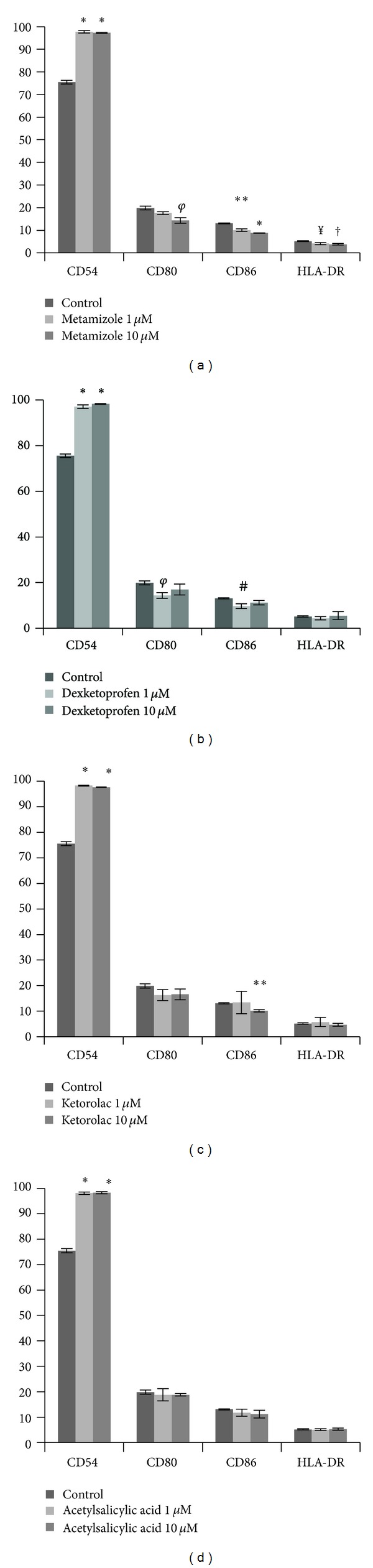
Percentage of expression of different surfaces markers of the osteoblast (MG-63), treated for 24 h with doses of 1 and 10 *μ*M of (a) metamizole, (b) dexketoprofen, (c) ketorolac, and (d) acetylsalicylic acid. *P < 0.01; **P = 0.001; ^*φ*^ *P* = 0.003; ^#^ *P* = 0.005; ^†^ *P* = 0.006; ^¥^ *P* = 0.03; ^‡^ *P* = 0.018.

The growth of control MG63 in nonosteogenic medium provided an antigenic profile typical of osteoblasts thus enabling a study of NSAID effect at therapeutic doses that could reflect that of primary osteoblasts. Indeed, flow cytometry showed that 75% of the control MG63 population expressed CD54, a cell adhesion protein highly expressed in osteoblast. The osteoblast phenotypic pattern was completed by the expression of CD80, CD86, and HLA-DR. These markers, shared by osteoblasts and immunocompetent cells, were all present in the cells albeit at a significantly lower levels. NSAIDs stimuli at both 1 and 10 *μ*M doses clearly altered these patterns of expression. Treatment with metamizole, dexketoprofen, ketorolac, and acetylsalicylic acid at 1 and 10 *μ*M doses for 24 h significantly increased the expression of CD54 above 90% of the whole cell population (*P* < 0.001). Flow cytometry results also showed that the modulation of the expression of CD80, CD86, and HLA-DR by the tested drugs depended on the type of NSAID used and their dosage. The incubation with 1 and 10 *μ*M of metamizole for 24 h significantly decreased the expression of CD80, CD86, and HLA-DR (Figure 1(a) and Table 2). However, dexketoprofen decreased the expression of CD80 and CD86 antigens only at doses of 1 *μ*M (*P* = 0.003 and *P* = 0.005, resp.) (Figure 1(b) and Table 2), and ketorolac decreased only the expression of CD86 (*P* < 0.001) at higher concentration (Figure 1(c) and Table 2). The treatment with acetylsalicylic acid at both doses did not modify the expression of CD80, CD86, and HLA-DR antigens (Figure 1(d) and Table 2).

### 3.2. Effect of NSAID on Phagocytic Activity

**Figure 2:**
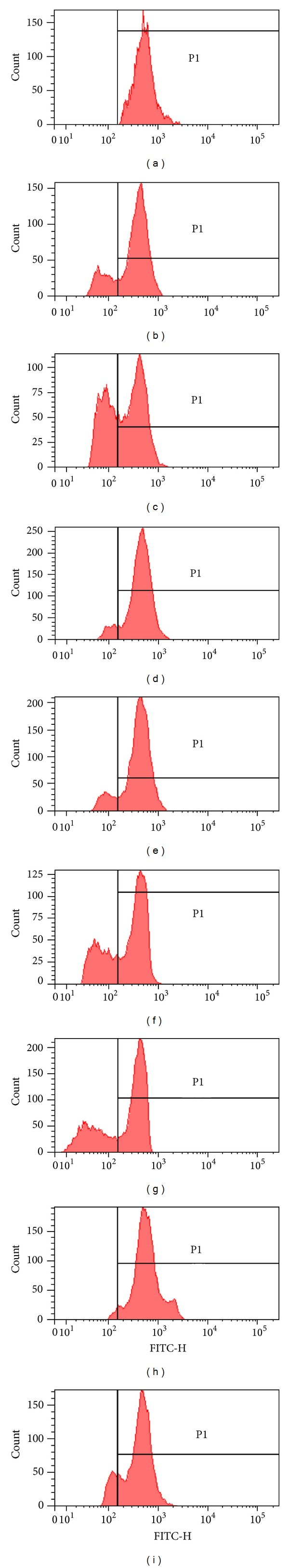
Fluorescence histogram of the phagocyte capacity of MG-63 cell line after treatment with different NSAIDs at doses of 1 and 10 *μ*M in comparison to control, determined by means of flow cytometry. (a) Control; (b) dexketoprofen 1 *μ*M; (c) dexketoprofen 10 *μ*M; (d) ketorolac 1 *μ*M; (e) ketorolac 10 *μ*M; (f) metamizole 1 *μ*M; (g) metamizole 10 *μ*M; (h) acetylsalicylic acid 1 *μ*M; (i) acetylsalicylic acid 10 *μ*M. These results are an example of one experiment.

The overall increase of markers of cell adhesion and reduced expression markers shared by the osteoblasts with immunocompetent cells prompted the study of their phagocytic activity. Consistently with the overall reduction of the immunocompetent markers, NSAIDs significantly reduced the phagocytic capacity of MG-63 cells, which was significantly decreased after a 24 h treatment with 1 and 10 *μ*M of dexketoprofen, ketorolac, and metamizole (*P* < 0.038). Only higher dosage acetylsalicylic acid led to a similar reduction of this specific cellular activity (*P* < 0.001) (Figure 2 and Table 3).

### 3.3. Effect of NSAID on Early MG63 Differentiation

The evidence of inhibition of the osteoblast immunocompetent phenotype led to the investigation of the effect of the NSAIDs on the ability of the cells to synthesise collagen, the main component of the osteoid, the immature extracellular matrix deposited during bone formation. This parameter of cell synthetic activity was associated to those of matrix mineralization that were studied through the early ALP specific activity and the later mineralized nodule formation. 

#### 3.3.1. Effect of NSAID on Collagen Synthesis

**Figure 3:**
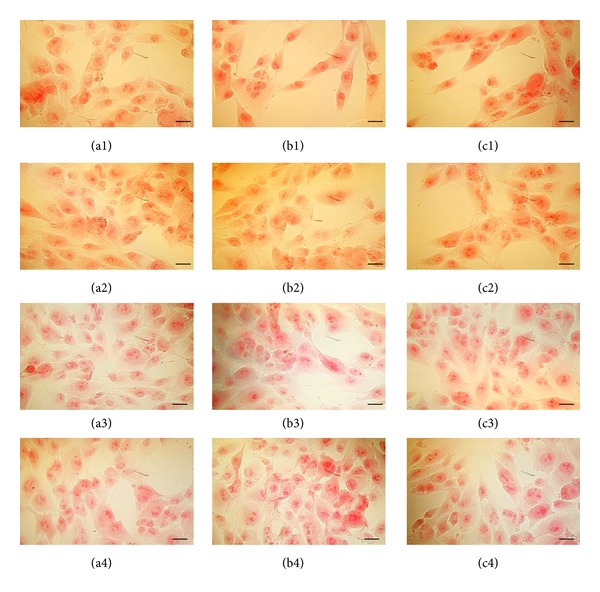
Pictures were taken under optical microscopy of the cells treated for 24 h with different NSAIDs ((1) dexketorpofen; (2) ketorolac; (3) metamizole; (4) acetylsalicylic acid) at different doses ((a) control; (b) 1 *μ*M; (c) 10 *μ*M) in nonosteogenic medium and dyed with Sirius red for to see the synthesis of collagen fibrins. Bars = 50 *μ*m.

**Figure 4:**
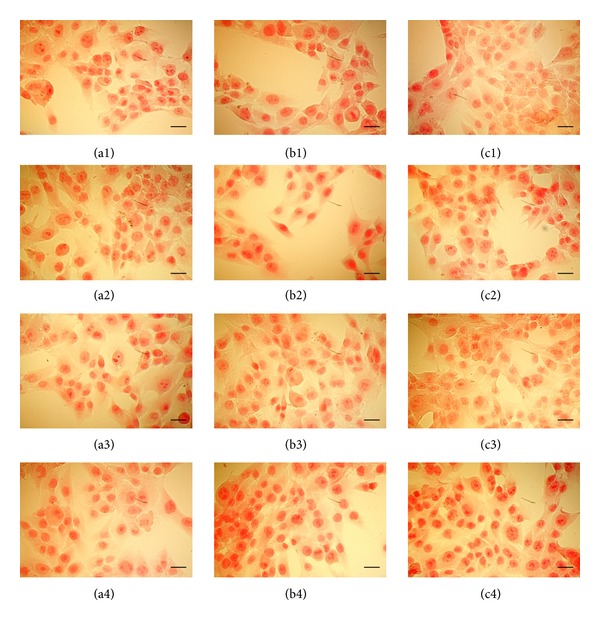
Pictures were taken under optical microscopy of the cells treated for 24 h with different NSAIDs ((1) dexketoprofen; (2) ketorolac; (3) metamizole; (4) acetylsalicylic acid) at different doses ((a) control; (b) 1 *μ*M; (c) 10 *μ*M) in osteogenic medium and dyed with Sirius red for to see the synthesis of collagen fibrins. Bars = 50 *μ*m.

**Figure 5:**
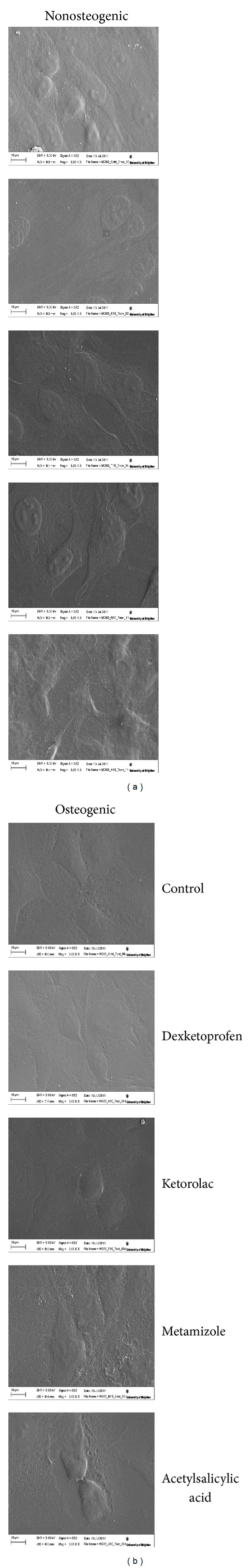
SEM: Effect of NSAIDs on collagen synthesis; cells were incubated in nonosteogenic and osteogenic mediums and treated at a dose of 10 *μ*M, for 7 days.

MG63 was not affected in its early ability to synthesise collagen. The qualitative analysis of collagen synthesis by MG-63 cells cultured in nonosteogenic and osteogenic media after 24 h incubation with the different drugs doses (1 and 10 *μ*M) (Figures 3 and 4) showed similar levels of positive staining mainly confined within the cytoplasm. SEM analysis clearly showed that the early synthesis of intracellular collagen fibrils detected by Sirius red was followed by secretion into the pericellular space after 7 days indicating a protected ability of these cells to produce extracellular matrix following drugs stimulation (Figures 5(a) and 5(b)).

#### 3.3.2. Effect of NSAIDs on MG63 Specific ALP Activity

The effect of the NSAIDs on the early biochemical pathway leading to extracellular matrix mineralization was studied by focusing on the assessment of the early levels of ALP activity. ALP measurements were performed on MG-63 cells cultured in osteogenic and nonosteogenic media, spiked with 1 and 10 *μ*M of dexketoprofen, ketorolac, metamizole, and acetylsalicylic acid. The results obtained are included in Tables 4(a) and 4(b) showing a significant decrease in the expression of this marker of differentiation when compared to control cells in all drug spiking and in nonosteogenic medium. However, when the experiment was performed in osteogenic medium a trend towards dose-dependent increase of the ALP specific activity was observed; statistical analysis was showing no significant difference.

#### 3.3.3. Effect of NSAID on Matrix Mineralization

The effect of the drug-reduced mineralization potential of the osteoid by the osteoblast ALP activity was observed by the late study of nodule mineralization.  Table 5 exhibits data on the number of red alizarin-stained mineralization nodules counted under light microscopy after 7, 15, and 20 days of culture in osteogenic medium. In control groups (untreated) at 7 days of culture, no nodules were observed in any well; at 15 days of culture, small nodules started to appear, and at 22 days of culture the number and size of nodules increased. Cells treated with dexketoprofen, ketorolac, metamizole, and acetylsalicylic acid (1 and 10 *μ*M) showed an inhibition in both the number and the size of nodules as compared to control group.

### 3.4. Effect of NSAID on MG63 Viability

**Figure 6:**
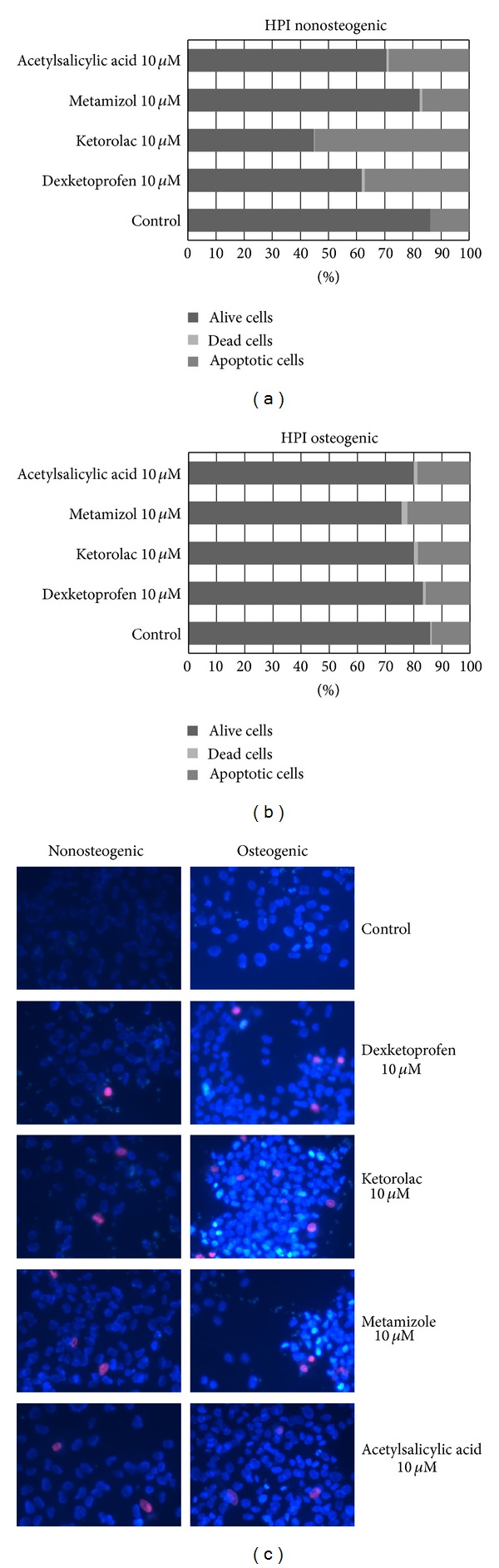
HPI: Effect of dexketoprofen, ketorolac, metamizole, and acetylsalicylic acid on cellular viability, cultured in nonosteogenic (a) and osteogenic mediums (b), after 24 h of treatment at a dose of 10 *μ*M. These micrographs are an example of MG63 treated with NSAIDs in nonosteogenic and osteogenic mediums (c).

MG63 viability was significantly reduced by the different NSAIDs when the drugs were tested at a 10 *μ*M dose in both nonosteogenic and osteogenic media after 24 h incubation (Figures 6(a) and 6(b)). HPI staining clearly showed an increase in apoptotic cells that were identified through the condensation of their chromatin appearing as bright staining by epifluorescence microscopy (Figure 6(c), arrows). The reduced cell viability appeared to depend on both the type of drug tested and the medium in which cells were cultured. When ketorolac and dexketoprofen were added in a nonosteogenic medium a significant increase of apoptotic cells was observed; metamizole and acetylsalicylic acid also increase the number of apoptotic cells but not significantly. While in osteogenic medium apoptosis was mainly stimulated ketorolac and metamizole compared with the control. The staining also showed a progression of the apoptotic process into necrosis; the process was appearing to be relatively faster in osteogenic medium (data not shown).

Osteoblasts are the most important cells in bone tissue; they play an important role in the processes of bone remodeling and formation. The growth, antigenic profile, and differentiation of osteoblasts are regulated by multiples local and systemic factors, which may regulate activity of a specific transcription factor [16, 26, 27]. These factors may show different effect on modulation of cellular metabolism depending on maturation and cell phenotype [28]. 

In this study, MG-63 cell line was used since they are recognized as cells the phenotype of which closely resembles that of primary osteoblasts. Indeed, in 2009 Díaz-Rodríguez et al. [22] described that this cell line has a similar antigenic prolife to that described in primary cultured human osteoblasts as in human bone tissue sections [21, 23]. The flow cytometry characterization of the control cells confirmed these findings validating the use of these cells for the study of the drug effect. Although with various patterns and efficacy all the tested drugs, but acetylsalicylic acid, appeared to inhibit the expression of the tested markers of expression for an immune phenotype and suggested an overall phenotypic pattern with an enhanced ability to adhere to the extracellular matrix, but as immature osteoblasts. 

The antigen CD54 express in a high percentage of MG63 cell line suffered a significant increase after 24 h of treatment with different NSAIDs. However, a slight decrease or no effect was observed in the expression of CD80, CD86, and HLA-DR. Acetylsalicylic acid showed no effect on the percentage of expression of any markers assayed. Dexketoprofen reduced CD80 and CD86 expression, and ketorolac decreased the percentage of expression on CD86. Only treatment with metamizole produced an inhibition of the three markers CD80, CD86, and HLA-DR at the two doses studied. 

Preosteoblasts and inmature osteoblasts express highly CD54 antigen [18], so its expression is related to the degree of cell differentiation and maturation. However, this expression can be modulated in presence of different biomolecules. Thus, it is known that primary cultures of human osteoblasts treatment with IL-1, IFN*γ*, or LPS increased the expression of CD54 antigen [27]. 

Osteoblasts and dendritic cells have characteristics in common including cytokine synthesis, phagocytic capacity, antigenic presentation to T lymphocytes, and the expression of certain antigens such as CD54, CD80, CD86, and HLA-DR [16, 19, 29]. The expression of these markers has been related to the degree of differentiation and/or cellular activation in both populations. Like us, other authors have studied the effect of acetylsalicylic acid on dendritic cells and found that immature dendritic cells treated with acetylsalicylic acid enhanced CD54 expression and decreased the expression of costimulatory molecules expression. These authors concluded that acetylsalicylic acid inhibits *in vitro* maturation and *in vivo* immunostimulatory function of murine dendritic cells [30, 31].

Dendritic cells are known to undergo two well-defined maturation stages, comprising immature dendritic cells and mature dendritic cells. Maturation of dendritic cells is associated with an increase of costimulatory molecules and with a more effective processing and presentation of antigens [29]. Comparative analysis of the two populations, osteoblasts and dendritic cells, suggests that the NSAIDs studied can inhibit osteoblasts differentiation and maturation. 

The treatment of MG-63 cell line with a higher dose (25 *μ*M) increased the expression of antigens CD21, CD44, CD80, CD86, and HLA-DR but not CD54 expression and decreased phagocytic activity [32]. However, this same research group found that the effect on primary human osteoblasts after treatment with ibuprofen did not change antigenic profile and phagocytic capacity [33]. These differences are attributed to different degrees of maturation of the osteoblasts in both studies.

The osteoblasts are cells that have a phagocytic capacity against different types and sizes of target particles [22]. The results of the present paper show that the treatment with dexketoprofen, ketorolac, metamizole, and acetylsalicylic acid decreases the phagocytic capacity of MG63 cell line in a dose-dependent manner. This effect would not be related to the behavior of dendritic cells because this phagocytic capacity decreases with maturation [29]. However, this is an effect commonly observed when the MG63 cell line has been treated with different NSAIDs [32, 34]. 

The treatment of MG-63 osteoblastic cells with dexketoprofen, ketorolac, metamizole, and acetylsalicylic acid, at therapeutic doses, led to no short-term effect on the ability of the cells to synthesise and deposit nonmineralized immature osteoid.

However, the biomineralization of the deposited collagenic extracellular matrix appeared to be inhibited unless osteogenic stimuli were provided to the cells. Osteoblasts differentiation and maturation evolve through a complex process regulated by multiple exogenous factors including growth factors, mechanical stimuli, or pharmacological treatments [31, 35–39]. Among the latter, works have been dedicated to unveiling the effect of various anti-inflammatory drugs. In particular, Abukawa and colleagues (2009) [39] found that high doses of ibuprofen (1 y 3 mmol/L) had a negative effect on differentiation of porcine osteoblastic cells finding suppression of ALP activity and decrease of mineralization matrix production. Also, short-term treatment with therapeutic doses of acetaminophen reduces cell proliferation and osteocalcin synthesis [34].

The inhibitory effect of NSAIDs on bone remodeling is also corroborated by the effect of the tested drugs on cell viability. The treatment of the MG-63 osteoblast-like cells with the various NSAIDs showed an increase in the percentage of apoptotic osteoblasts lately developing into necrotic cells. It can be speculated that this reduced cell viability would alter remodeling with osteoblasts experiencing a reduced viability and ability to mineralize the deposited extracellular matrix.

## 4. Conclusions

The present studies have applied an *in vitro* cell culture model based on osteosarcoma MG-63 osteoblast-like cells to study the effect of therapeutic concentrations of several types of NSAIDs. The choice of markers of expression and phenotypic differentiation at short and prolonged time of exposure of the cells to the drugs has allowed to unveil that, although with differences, this class of anti-inflammatory substances can alter bone remodeling by reducing cell maturation, its longevity and biochemical machinery necessary to mineralize the deposited extracellular matrix. It may therefore be speculated that their protracted use could indeed lead to pathological conditions such as osteoporosis.

## Figures and Tables

**Table 1:** Monoclonal antibodies (MABs) used to study the effect of dexketorpofen, ketorolac, metamizole, and acetylsalicylic acid on antigenic phenotype of cultured MG63 osteoblastic-like cells, showing their specificity, and the fluorochrome used to label the antibody and the supplier.

MABs	CD/specificity	Fluorochrome	Supplier
CONTROL	—	FITC	Invitrogen Corp., (Carlsbad, CA, USA)
CD54/IOL1b	CD54	FITC	Invitrogen Corp., (Carlsbad, CA, USA)
CD80	CD80	FITC	Invitrogen Corp., (Carlsbad, CA, USA)
CD86	CD86	FITC	Invitrogen Corp., (Carlsbad, CA, USA)
OKDR	HLA-DR	FITC	Invitrogen Corp., (Carlsbad, CA, USA)

FITC: fluorescein isothiocyanate.

**Table 2:** Percentage of expression (by flow cytometry) of different antigens expressed in MG63 cell line after 24 h of treatment with different anti-inflammatories, at doses of 1 and 10 *μ*M.

Treatment	Monoclonal antibodies							
	CD54		CD80		CD86		HLA-DR	
	%, SD^a^	*P*	%, SD^a^	*P*	%, SD^a^	*P*	%, SD^a^	*P*
Control	75.5 (0.83)	—	19.9 (0.83)	—	13.1 (0.20)	—	5.2 (0.26)	—
Metamizole 1 *µ*M	97.7 (0.57)	0.00*	17.6 (0.61)	0.018*	10.1 (0.6)	0.001*	4.13 (0.49)	0.03*
Metamizole 10 *µ*M	97.3 (0.2)	0.00*	14.4 (1.24)	0.003*	8.8 (0.1)	0.00*	3.8 (0.36)	0.006*
Dexketoprofen 1 *µ*M	97 (0.8)	0.00*	14.4 (1.24)	0.003*	9.7 (1.05)	0.005*	4.43 (0.75)	0.17
Dexketoprofen 10 *µ*M	98.2 (0.15)	0.00*	17 (2.36)	0.11	11.2 (0.96)	0.06	5.56 (1.74)	0.73
Ketorolac 1 *µ*M	98.2 (0.17)	0.00*	16.3 (2.13)	0.11	13.43 (4.38)	0.926	5.76 (1.77)	0.63
Ketorolac 10 *µ*M	97.6 (0.1)	0.00*	16.6 (2.13)	0.055	10.2 (0.45)	0.001*	4.73 (0.58)	0.27
Acetylsalicylic acid 1 *µ*M	98.1 (0.5)	0.00*	18.8 (2.45)	0.51	11.8 (1.37)	0.24	5.06 (0.35)	0.627
Acetylsalicylic acid 10 *µ*M	98.3 (0.36)	0.00*	18.8 (0.45)	0.12	11.2 (1.49)	0.09	5.26 (0.46)	0.839

^a^Standard deviation, *significant differences *P* < 0.05.

Bold values is the statistical significance.

**Table 3:** Percentage of cells with phagocyte capacity determinate by flow cytometry; treated with metamizole, dexketoprofen, ketorolac, and acetylsalicylic acid at doses of 1 and 10 *μ*M.

Treatment	% of cells phagocytes (mean value)	SD^a^	*P* value
Control	98.89	1.5217	
Metamizole 1 *µ*M	78.20	1.9287	0.000*
Metamizole 10 *µ*M	65.10	11.8528	0.038*
Dexketoprofen 1 *µ*M	90.39	2.3180	0.001*
Dexketoprofen 10 *µ*M	87.36	1.5534	0.001*
Ketorolac 1 *µ*M	68.93	7.2037	0.017*
Ketorolac 10 *µ*M	62.96	5.9877	0.007*
Acetylsalicylic Acid 1 *µ*M	96.29	1.7235	0.035
Acetylsalicylic Acid 10 *µ*M	88.47	1.9612	0.000*

^a^Standard deviation, *significant differences *P* < 0.05.

**Table:** (a)

Treatment	Mean	SD^a^	*P* value
Control	2.67	0.117	
Dexketoprofen 1 *µ*M	1.77	0.253	0.005*
Dexketoprofen 10 *µ*M	1.88	0.207	0.005*
Ketorolac 1 *µ*M	1.65	0.338	0.008*
Ketorolac 10 *µ*M	1.79	0.184	0.002*
Metamizole 1 *µ*M	1.69	0.030	0.000*
Metamizole 10 *µ*M	1.92	0.083	0.001*
Acetylsalicylic acid 1 *µ*M	1.77	0.137	0.001*
Acetylsalicylic acid 10 *µ*M	1.95	0.173	0.005*

^a^Standard deviation, *significant differences *P* < 0.05.

**Table:** (b)

Treatment	Mean	SD^a^	*P* value
Control	2.37	0.092	
Dexketoprofen 1 *µ*M	2.44	0.293	0.718
Dexketoprofen 10 *µ*M	2.76	0.392	0.165
Ketorolac 1 *µ*M	2.68	1.772	0.029
Ketorolac 10 *µ*M	3.01	3.522	0.216
Metamizole 1 *µ*M	2.43	0.319	0.775
Metamizole 10 *µ*M	3.70	1.602	0.223
Acetylsalicylic acid 1 *µ*M	2.54	1.878	0.446
Acetylsalicylic acid 10 *µ*M	3.31	0.478	0.602

^a^Standard deviation.

**Table 5:** Qualitative study of mineralization of MG63 cell line through formation of nodules, in relation to time, cultured in osteogenic medium supplemented with different NSAIDs: dexketoprofen, ketorolac, metamizole, and acetylsalicylic acid (1 and 10 *μ*M).

Treatment	7 days		15 days		20 days	
	Number of nodules	Size of nodules	Number of nodules	Size of nodules	Number of nodules	Size of nodules
Control	−	−	+	40–80 *µ*m	++	80–150 *µ*m
Acetylsalicylic acid 1 *µ*M	−	−	±	10–40 *µ*m	+	50–100 *µ*m
Acetylsalicylic acid 10 *µ*M	−	−	±	10–40 *µ*m	+	50–100 *µ*m
Dexketoprofen 1 *µ*M	−	−	−	−	+	50–100 *µ*m
Dexketoprofen 10 *µ*M	−	−	−	−	−	−
Ketorolac 1 *µ*M	−	−	−	−	+	40–80 *µ*m
Ketorolac 10 *µ*M	−	−	−	−	+	40–80 *µ*m
Metamizole 1 *µ*M	−	−	−	−	+	10–40 *µ*m
Metamizole 10 *µ*M	−	−	−	−	−	−

− No detected nodules; ± of 0 to 10 nodules per well; + of 5 to 20 nodules per well; ++ more than 20 nodules per well.
